# Impact of dental aesthetics on psychosocial well-being: A cross-sectional study

**DOI:** 10.6026/973206300220061

**Published:** 2026-01-31

**Authors:** Irish Guangchuiliu Pamei, Anil Sharma, Omar Mukhtar, Sanatomba Thingujam, Karanjyot Kaur, Sumanta Saikia

**Affiliations:** 1 Department of Prosthodontics and Crown & Bridge, Kalka Dental College, Meerut, Uttar Pradesh, India

**Keywords:** Dental aesthetics, dental confidence, psychosocial impact, self-esteem, treatment awareness

## Abstract

The lack of awareness about dental treatments and gender differences in aesthetic preferences affect the relationship between dental aesthetics, psychosocial well-being. Therefore, it is of interest to enhance understanding of the relationship between dental aesthetics and psychosocial well-being, and dental confidence among non-dental students, highlighting gender differences and awareness gaps. Hence, a total of 400 students were surveyed using the Psychosocial Impact of Dental Aesthetics Questionnaire (PIDAQ). The results highlight the significant influence of dental appearance on self-esteem, social interaction, and mental health. A greater desire for dental aesthetic improvement was observed among females, and there was a notable lack of awareness about available dental treatments. Thus, we show the impact of dental aesthetics on psychosocial well-being and dental confidence among non-dental students at Kalka Dental College, Meerut, with a focus on awareness and gender differences.

## Background:

The human face has long been regarded as a reflection of identity, emotion, and individuality. It serves not only as a medium of expression but also as a powerful contributor to a person's self-perception and social presence [1]. Among its various components, the smile holds particular significance, as it is central to communication, aesthetics, and psychosocial well-being [2]. The term aesthetics, derived from the Greek word aesthesis meaning "sensuous knowledge,"originally emerged in the eighteenth century to distinguish sensory perception from logical reasoning. In dentistry, aesthetics encompasses the principles of beauty and harmony as applied to the oral and maxillofacial region. With growing societal emphasis on appearance and self-presentation, dental aesthetics have become increasingly important in shaping an individual's confidence, self-image, and social interactions [3]. Aesthetic dental concerns extend beyond visual appeal; they carry profound psychological and emotional consequences. The appearance of teeth and smile can influence a person's self-esteem, social comfort, and quality of life. Individuals with dental irregularities or perceived unattractive teeth often report feelings of embarrassment, lowered self-confidence, and avoidance of social situations [4]. Traditionally, dental research placed greater emphasis on clinical evaluation of malocclusion and dental aesthetics through indices such as the Dental Aesthetic Index (DAI) and the Aesthetic Component of the Index of Orthodontic Treatment Need (IOTN-AC). While these tools offer objective assessment, they do not fully capture the subjective psychosocial burden experienced by individuals [5]. To address this gap, health-related quality of life (HRQoL) tools such as the Psychosocial Impact of Dental Aesthetics Questionnaire (PIDAQ) have been developed. PIDAQ offers comprehensive insight into emotional well-being, social impact, dental self-confidence, and aesthetic concern [6]. Such tools highlight that dental aesthetics are closely linked to mental health outcomes, including self-esteem, social anxiety, and psychological distress [7].

Young adults, including high school and college students, are particularly vulnerable to the psychosocial impact of poor dental aesthetics. This age group is highly conscious of appearance, influenced by peer perceptions, and increasingly shaped by social media culture [8]. Unattractive teeth resulting from malocclusion, dental trauma, caries, or harmful oral habits can significantly affect their social interactions, confidence levels, and willingness to seek dental care. In modern society- where digital visibility and social engagement are heightened-the pressure to maintain an aesthetically pleasing smile is stronger than ever [9]. Awareness of dental treatment options plays a crucial role in shaping dental confidence and motivating individuals to pursue care. When people are informed about available aesthetic and corrective procedures, they are more likely to seek professional help, overcome self-consciousness, and develop a positive attitude toward oral health. Increasing awareness also fosters a supportive, stigma-free environment where aesthetic dental treatments are viewed as legitimate and empowering choices [10]. Improved dental confidence following aesthetic enhancement often leads to greater self-esteem, better social participation, and improved psychological resilience [11]. Therefore, it is of interest to determine the awareness, perceived satisfaction, psychosocial impact, and dental confidence associated with dental aesthetics among students.

## Methodology:

The present study was conducted among students from various non-dental courses enrolled at the Kalka Group of Institutions, Meerut, Uttar Pradesh. A total sample of 400 participants, consisting of 200 males and 200 females, was selected. The sample size was determined using G*Power software version 3.1.9.6, developed by Franz Faul, University of Kiel. Using a 95% confidence interval, a 5% allowable error, and a 50% response distribution with a Z-value of 1.96, the minimum required sample size was calculated to be approximately 380, which was rounded to 400 for convenience and to increase reliability. A cross-sectional survey design was employed to assess dental aesthetics, psychosocial impact, awareness, and dental confidence among non-dental students. Both quantitative and qualitative elements were incorporated into the study design. Data were collected through a structured questionnaire distributed among students. Before completing the questionnaire, participants were informed about the objective of the study and the procedures involved. The questionnaire consisted of items derived from the PIDAQ, assessing domains such as dental self-confidence, aesthetic concern, social impact, and psychosocial influence. Participants evaluated each item using a three-point Likert scale, with responses coded as 0 for "No,"1 for "Maybe,"and 2 for "Yes. "The questionnaire also included items assessing satisfaction with smile aesthetics, willingness to visit a dentist, awareness of treatable dental conditions, and perceptions related to their dental appearance. Since the study was conducted within a single institutional campus, data collection was convenient and efficient. All participants demonstrated adequate comprehension of the questions, and the questionnaire format was easy to understand. The inclusion criteria restricted the study to students aged 17-28 years enrolled in non-dental programs at the institution. Dental students were excluded to avoid bias due to prior knowledge or professional exposure. Individuals below 16 or above 29 years of age were also excluded. Participants with a history of dental aesthetic treatment, such as orthodontic therapy or smile design procedures, were removed from the study to ensure that responses reflected natural perceptions rather than post-treatment outcomes. Additionally, any questionnaire that was incomplete or improperly filled out was discarded based on discontinuation criteria. The finalized data were entered into Microsoft Excel 2007 and subsequently analyzed using SPSS version 23.0 statistical software. Descriptive statistics, including frequency and percentage distributions, were used to summarize participant responses. The Chi-square test was applied to assess associations between categorical variables, and the level of statistical significance was set at 5% (p <0.05). The Chi-square test, which compares observed values to expected values under a given hypothesis, was particularly useful for determining whether differences in responses were statistically meaningful. A large Chi-square value indicated a greater deviation between observed and expected frequencies, suggesting that the variables under study may be significantly associated.

## Results:

**Figure 1:**
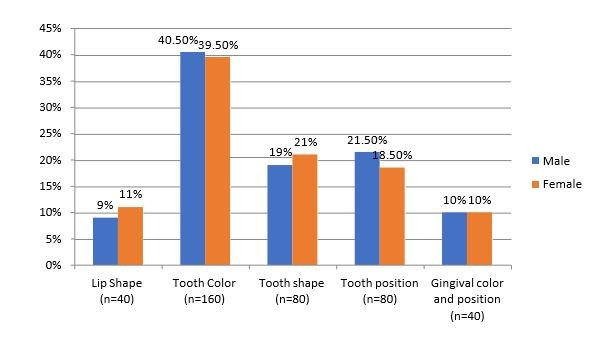
The percentage and its graphical representation based on the factors of dissatisfaction among the subjects

**Figure 2:**
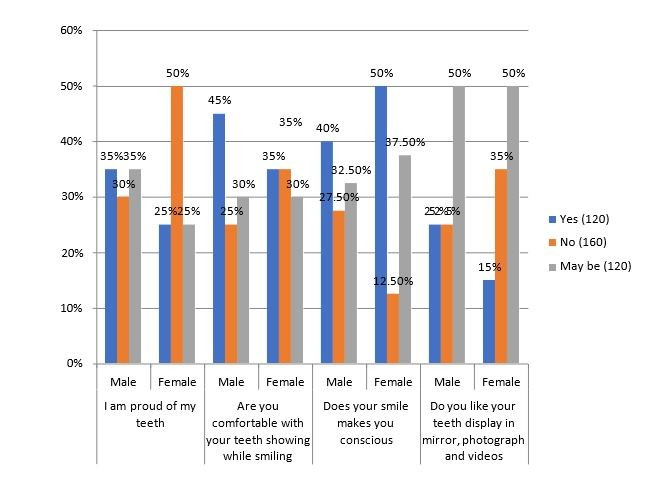
The percentage and its graphical representation based on the dental self-confidence

**Figure 3:**
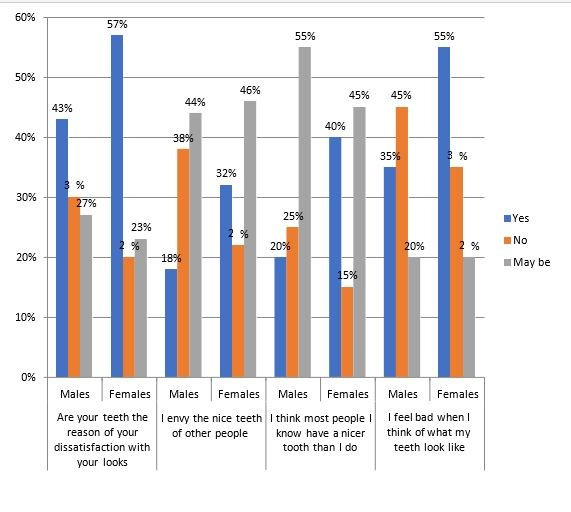
The percentage and graphical representation based on psycho- social impact

Out of 400 total individuals, 200 males (or 50%) and 200 females (or 50%) make up each slice, which is sized to correspond to their proportion to the total (Table 1). Of the two hundred males, twenty-two are extremely satisfied (11%) and 72 are somewhat content (36%); 106 are not happy (53%). Of the 200 women in total, 18 are extremely happy with 9%, 48 are content with 24%, and 134 are not happy with 67% displaying a non-significant Chi Square test with a p value greater than 0.05. Figure 1 displays the factors that contribute to dissatisfaction. Of the respondents, 18 males (9%) and 22 females (11%), indicated that the lip shape was the source of their dissatisfaction; 81 males (40.55%) and 79 females (39.5%) indicated that their discontent stemmed from the tooth colour; 38 males (19%) and 42 females (21%) from the tooth shape; 43 males (21.5%) and 37 females (18.5%)indicated that the tooth position were the sources of their dissatisfaction; 20 males (10%) and 20 females (10%) with the Chi Square test p value greater than 0.05 is non-significant. Figure 2 shows the tabular and graphical distribution on dental self-confidence. Among the male participants, 35% indicate feeling proud of their teeth, while 30% express a lack of pride, and 35% remain ambivalent. In contrast, the female respondents exhibit a slightly lower proportion (25%) expressing pride in their teeth, with 50% indicating a lack of pride and 25% remaining uncertain with statistical significance of 0.004 .45% male participants, express comfort with displaying their teeth while smiling, while 25% indicate discomfort, and 30% remain uncertain. Meanwhile, 35% of female respondents express comfort, 35% indicate discomfort, and 30% remain uncertain. With a non-significant value of .071 and 50% of both male and female participants feel conscious about their smiles, while 27.5% of male participants and 12.5% of female participants do not experience such consciousness with a significant value of 0.001. Figure 3 shows the psycho-social impact data. Female participants (57%) attribute their dissatisfaction with their looks to their teeth compared to male participants (43%) with significant value of 0. 020.32% female express envy for the nice teeth of others compared to male participants (18%) with a significant value of 0.004. Also, female participants (40%) believe that most people they know have nicer teeth than they do, as compared to male participants (20%) with a significant value of 0.001. And 55% females express feeling bad when thinking about what their teeth look like compared to male participants (35%) 0.05, which is a noteworthy value.

**Figure 4:**
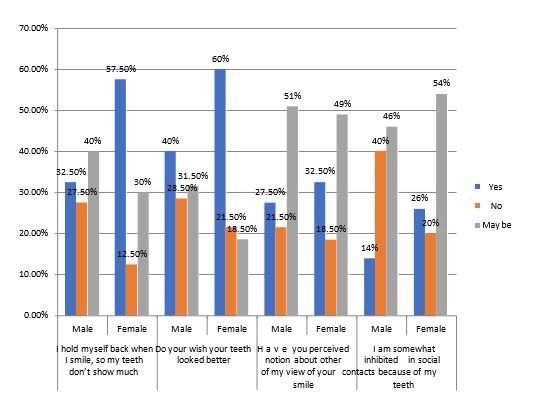
The percentage and its graphical representation based on social impact among study subjects

**Figure 5:**
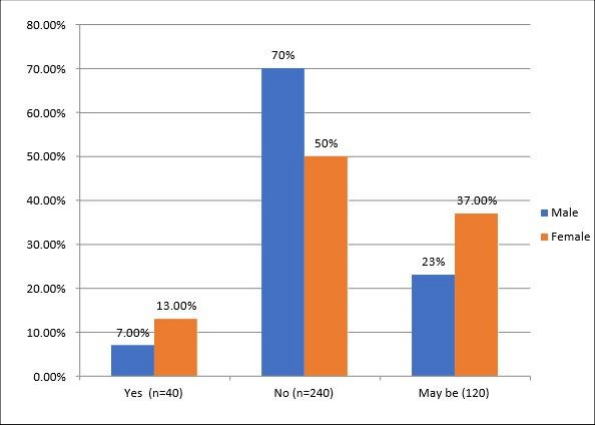
The percentage and its graphical representation based on the knowledge of possibilities of treatment

Figure 4 displays the data on the social impact among the study subjects of 400 young adults, 32.5% male participants, indicate that they hold themselves back when they smile, 27.5% assert that they do not, and 40% remain uncertain. 57.5% of female respondents indicate that they hold themselves back when they smile, 12.5% assert that they do not, and 30% remain uncertain. 40% male participants express a wish for their teeth to look better, 28.5% indicate no such wish, and 31.5% remain uncertain. 60% of female participants express a want for their teeth to look better, 21.5% indicate no such wish, and 18.5% remain uncertain.27.5% male participants, indicate having perceived notions about others' views of their smiles, 21.5% indicate no such perceptions, and 51% remain uncertain. 32.5% of female respondents indicate having perceived notions, 18.5% indicate no such perceptions, and 49% remain uncertain. Among male participants, 14% indicate feeling inhibited, 40% indicate no inhibition, and 46% remain uncertain. For female participants, 26% indicate feeling inhibited, 20% indicate no inhibition, and 54% remain uncertain. Based on their concerns about their teeth, both male and female participants showed readiness to visit a dentist. Female participants expressed a higher willingness to visit the dentist than male participants (55% vs. 45%), with a significant value of 0.057. In Figure 5, the awareness of treatment options between male and female participants is depicted. Of the male participants, 14 (7.0%) claim to know, 140 (70%) do not know, and 46 (23%) are unsure. With a significant value of 0.004, 26 (13.0%) females assert to know, 100 (50%) females have no knowledge, and 74(37.0%) females are uncertain.

## Discussion:

In the present campus-based cross-sectional study, a substantial proportion of non-dental students reported concerns about their dental appearance and acknowledged that their teeth influenced their psychosocial well-being and dental self-confidence. Consistent with earlier work, domains such as aesthetic concern, social impact and dental self-confidence emerged as key areas through which dental appearance affected day-to-day life. These findings reinforce the view that dental aesthetics are not merely a cosmetic issue but are closely tied to self-perception, willingness to smile openly, and comfort in social interactions. Our results are broadly comparable with those of Afroz *et al.* who investigated North Indian university students and reported that although most participants were satisfied with their smile, tooth colour and other aesthetic factors still contributed appreciably to dissatisfaction and influenced social behaviour and dental self-confidence. Like Afroz *et al.* (2013) [4] study, the present work also suggests that even in a relatively young and educated population, subtle aesthetic deviations are enough to make some students hide their teeth while smiling, feel self-conscious in photographs, or hesitate to seek social contact. Stojilkovic *et al.* (2024) [11] assessed Nigerian university undergraduates using PIDAQ and demonstrated that impaired dental aesthetics most strongly affected the dental self-confidence subscale, with clear differences in psychosocial well-being across levels of perceived malocclusion. The pattern observed in our sample, where perceived dental defects notably influenced dental self-confidence and social interaction, is in line with these findings and supports the utility of PIDAQ-derived constructs in capturing student experiences across different cultural contexts. The negative association between poorer perceived dental aesthetics and self-esteem observed in our participants mirrors the relationships described by Venete *et al.* [12], who reported a significant negative correlation between total PIDAQ scores and Rosenberg self-esteem among university students in Spain, as well as a positive association with perfectionism. Their work suggested that students more affected by dental aesthetics tend to have lower self-esteem and higher perfectionist traits; our findings similarly indicate that dissatisfaction with smile and teeth may contribute to heightened self-consciousness, worry about others' opinions, and avoidance of smiling, although personality traits were not formally assessed in our study. Taken together, our findings, in conjunction with these four studies, underscore that dental aesthetics exert a consistent psychosocial impact on young adults across different settings. This supports the need for campus-based awareness initiatives, accessible counselling, and integration of information on aesthetic treatment options into oral health promotion so that students can make informed decisions and improve both their dental confidence and overall psychosocial well-being.

## Conclusion:

Dental aesthetics significantly influence students' psychosocial well-being, social interactions, and dental self-confidence. Limited awareness of available aesthetic treatments further contributes to dissatisfaction and hesitancy in seeking care. Strengthening dental education and promoting awareness can enhance students' confidence and support better oral health decisions.

## Figures and Tables

**Table 1:** Gender distribution among study subjects

	**N**	**Percentage**
Male	200	50%
Female	200	50%
